# Show cards of the Global Physical Activity Questionnaire (GPAQ) – do they impact validity? A crossover study

**DOI:** 10.1186/s12889-020-8312-x

**Published:** 2020-02-12

**Authors:** Kevin Rudolf, Florian Lammer, Gerrit Stassen, Ingo Froböse, Andrea Schaller

**Affiliations:** 1 Institute of Movement Therapy and movement-oriented Prevention and Rehabilitation, German Sport University Cologne, Am Sportpark Müngersdorf 6, 50933 Cologne, Germany; 2 Working group physical activity-related prevention research, German Sport University Cologne, Am Sportpark Müngersdorf 6, 50933 Cologne, Germany; 3 Center for Health and Physical Activity, German Sport University Cologne, Am Sportpark Müngersdorf 6, 50933 Cologne, Germany; 4 IST-University of Applied Sciences, Erkrather Straße 220 a-c, 40233 Düsseldorf, Germany

**Keywords:** Physical activity assessment, Accelerometry, Instrument psychometrics, Measurement, Methods

## Abstract

**Background:**

The Global Physical Activity Questionnaire (GPAQ) is applied internationally as a tool to assess the level of physical activity. The GPAQ was designed as an interview, including the use of show cards, which visualise activities of moderate and intensive physical activity and support the distinction between these intensities. The self-administered version of the GPAQ is used in the application-oriented research for reasons of economy and practicality. However, the use of show cards often remains unknown.

The aim of the present study was to examine differences in validity between two self-administered versions of the GPAQ with and without show cards.

**Methods:**

In this crossover study, two groups (*n* = 54; 57.4% female; 28.3 ± 12.2 years) received the GPAQ with or without show cards after 7 days and the respective other version after additional 7 days. For validation, all participants wore an accelerometer (ActiGraph GT3X+) on all 14 days.

Differences between GPAQ versions and accelerometer data were compared by Wilcoxon signed rank test. Additionally, Spearman analyses and Bland-Altman plots were calculated.

**Results:**

No statistically significant difference between the GPAQ versions could be found in regard to the accuracy of physical activity assessment (*p* > 0.05).

Both GPAQ versions show similar correlation coefficients for vigorous physical activity (rho = 0.31–0.42) and sedentary behaviour (rho = 0.29–0.32). No statistically significant correlation was found for physical activity of moderate intensity. The Bland-Altman plots support these results, as both GPAQ versions have the same trends in terms of overestimation and underestimation of physical activity.

**Conclusion:**

The use of show cards had no significant impact on questionnaire validity. Therefore, both GPAQ versions can be applied interchangeably. Nevertheless the exact description of application of the GPAQ is desirable in terms of reproducibility and transparent scientific research.

## Background

Even though physical activity is widely recognized as a cornerstone of a healthy lifestyle and many campaigns and interventions aim to promote physical activity, the measurement of physical activity including the scientific evaluation of these programs remains a challenge.

Over the last decades a large variety of instruments to assess physical activity emerged [1–5]. Whereas the doubly-labeled water method (DLW) [6] relies on the chemical measures of energy expenditure, others, like accelerometers, record proper accelerations, and still others, like questionnaires, focus on the individual’s memory and perception of physical activity.

This diversity of measurement techniques inevitably comes along with different assets and drawbacks regarding validity and feasibility [1, 2, 4, 5]. While the DLW represents high validity in assessing total energy expenditure, it is a complex and expensive procedure that provides a total value of energy expenditure for a certain time frame but does not give information, e.g., on several individual periods of time or the context of energy expenditure [3, 5, 7]. Questionnaires, on the other side, are considered a more practicable tool to assess physical activity in large samples [2, 5]. Although, disadvantages in its utility are apparent, such as the inaccuracy due to lack of memory, social desirability and other social and cognitive factors [8–10], they can provide insight into the context of physical activity as well as retrospective data over various time frames [1, 11]. Moreover, they are usually simple to administer, easy to distribute online and inexpensive [3, 5, 12]. Therefore questionnaires are often the method of choice for practice-oriented research [13].

In 2002, the World Health Organization (WHO) initialized its STEPwise approach for surveillance of risk factors for chronic disease (STEPS). In line with STEPS, the Global Physical Activity Questionnaire (GPAQ) was designed to assess physical activity in different settings and cultures around the world. According to the WHO website (https://www.who.int/ncds/surveillance/steps/GPAQ/en/), the GPAQ has been used in more than 100 countries to assess physical activity at work, in leisure time and for transportation purposes. It differentiates between moderate and vigorous physical activity and has an additional question regarding daily sedentary behaviour [14].

In its original version, the GPAQ was designed as an interview which has to be adapted to the respective culture in which it is applied [14]. Previous validation studies showed acceptable reliability [15, 16] and validity [15, 17] for the GPAQ in various countries. Moreover, self-administered versions of the GPAQ have been validated over the past years showing comparable quality criteria [18, 19].

However, the original version of the GPAQ includes so called “show cards”, which are adapted to illustrate exemplary physical activities of moderate and vigorous intensity typical for the respective culture [14]. In many studies that use the GPAQ for the assessment of physical activity, it remains unclear whether they use these show cards, though [20–22]. Hence, the question arises whether the validity of the GPAQ changes depending on the application of show cards.

### Objective

The present study aims to examine differences regarding validity between two self-administered versions of the GPAQ with and without show cards.

## Methods

### Study design

**Fig. 1:**
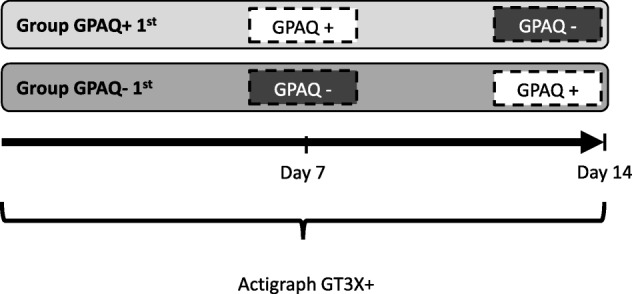
Study design. Group GPAQ+ 1st received the GPAQ with show cards (GPAQ+) after the first week and the GPAQ without show cards (GPAQ-) after week two. The procedure for Group GPAQ- 1st was the other way around

The current study was conducted as a randomized crossover trial. Participants were randomized to answer the German version of the GPAQ with show cards depicting examples of moderate and vigorous physical activities (“GPAQ+”) or without such show cards (“GPAQ-“) after 7 days. After the next 7 days, the two study groups were reversed according to the randomized crossover design (see Fig. 1). In addition, objective physical activity data of all participants were recorded by accelerometry (ActiGraph GT3X+) over the whole investigation period of 14 days (2 × 7 days).

### Participants

A convenience sample was recruited mainly via bulletins at the German Sport University Cologne and different sport clubs in Cologne, Germany. Interested individuals were contacted via email or telephone and given detailed instructions about the study.

Recruitment and data collection were conducted between May and July 2017. Recruitment was closed after more than 50 usable datasets were collected [23].

#### Eligibility criteria

Inclusion criteria were the participants’ age of at least 18 years, understanding German, and access to the internet on day seven and day 14 to answer the respective version of the GPAQ online. Individuals with acute musculoskeletal diseases or orthopaedic injuries were excluded.

The study was approved by the ethical committee of German Sport University Cologne (reference: 084/2017). Participants received a report on their individual accelerometer measured physical activity after the end of the study. No other incentives for participating in the study were provided. All participants provided written informed consent.

### Measures

#### Global Physical Activity Questionnaire (GPAQ)

The German version of the GPAQ (available at https://www.who.int/ncds/surveillance/steps/GPAQ/en/) was adapted to a self-administration format by rephrasing the first paragraph, which includes sentences only appropriate in an interview [18]. Consistent with the instruction manual of the GPAQ [24], culturally adapted examples of different physical activities were inserted in the respective questions.

For this study, the GPAQ was delivered in two different versions. GPAQ+ included pictures of work or leisure time activities of moderate or vigorous intensity, while GPAQ- included no pictures. In line with the instruction manual calling for cultural adaptation [24], pictures in GPAQ+ were selected to represent typical physical activities of the German population. In order to match the pictures to the respective intensities, the” Compendium of Physical Activities “[25] was used. Activities with 2–6 MET (e.g., working as a mailman, Nordic walking, yoga) were considered moderate intensity, activities with more than 6 MET were considered vigorous intensity (e.g., working as a construction worker, running, basketball). For sedentary behaviour – in line with the instruction manual - no pictures were included in either version. The resulting show cards can be seen in Additional file 1.

For practical reasons, the two versions of the GPAQ were administered via the online survey tool Unipark (Questback GmbH, Cologne, Germany). In this way, transcription errors were avoided and data were automatically available in digital form. A link to the respective GPAQ version was automatically sent by the system to the participants’ individual email-addresses at the morning of day 7 and day 14. Reminder emails were automatically sent if participants did not answer the questionnaire within 2 days after receiving the link.

#### Actigraph GT3X+

Objective physical activity was recorded over 14 days with Actigraph GT3X+ accelerometers (ActiGraph, Pensacola, Florida, USA). This type of accelerometer has shown valid results for the assessment of physical activity in adults [26–29].

Participants were instructed to wear the accelerometer on the right-side of their waist for 14 consecutive days, removing them only whilst sleeping or participating in any water-based activity (e.g., showering or swimming). A picture of the application of the accelerometer can be seen in Additional file 2.

Data was collected with a sample rate of 30 Hz and saved in 30-s epochs.

In line with initializing and handing out the accelerometers, demographic variables (sex, age, body mass index) of all participants were recorded.

### Statistical analyses

#### Data preparation

GPAQ data were cleaned according to the GPAQ analysis guide [24]. In a next step, daily averages (minutes/day) were calculated for moderate and vigorous physical activity, respectively. For this purpose, physical activity at work, in leisure time and transportation were combined. This procedure was applied to both GPAQ versions, resulting in daily averages of moderate and vigorous physical activity for each version.

The accelerometer data were processed using the ActiLife software (version 6.10.2, ActiGraph, Pensacola, Florida, USA).

Since the data were recorded as counts per minute (CPM), the following cut-points [30] were applied to classify CPM into intensities of physical activity:

- 0–99 CPM: sedentary behaviour,
- 100–1951 CPM: light intensity,
- 1952–5724 CPM: moderate intensity,
- > 5724 CPM: vigorous intensity.

Sixty or more minutes of consecutive zero CPM were marked as non-wear time (allowing for an interruption of maximum 2 minutes with 1–100 CPM) [31]. Individuals were included in the analysis if valid data of at least 3 days with a minimum of 10 hours wear time were available for each of the 2 weeks.

In line with the processing of the GPAQ data, daily averages (min/day) of valid days were calculated for moderate and vigorous physical activity (minutes in each activity category divided by the number of recorded days). Data of week one and two were handled separately.

#### Data analyses

All statistical analyses were conducted using IBM SPSS Statistics 24.

Descriptive statistics (means, standard deviations, frequencies and percentages) were used to describe demographic characteristics and the data from questionnaires and accelerometers. Chi-squared tests and Mann-Whitney U tests were used to examine statistically significant differences between groups at baseline and between the included and excluded sample.

Statistical significant differences regarding the validity of the two GPAQ versions were examined using Wilcoxon signed-rank tests. For this purpose, the differences of each GPAQ version to the accelerometer data were compared (i.e., GPAQ+ data minus accelerometer data, and GPAQ- data minus accelerometer data).

Spearman’s rank correlation coefficients were calculated for the correlation between the daily averages of the accelerometer data and the data from each GPAQ version. The resulting coefficients were interpreted as no correlation (rho = 0–0.09), poor (rho = 0.10–0.29), fair (rho = 0.30–0.59), moderately strong (rho = 0.6–0.79), very strong (rho = 0.8–0.99), and perfect correlation (rho = 1) [32].

In addition, Bland-Altman plots were built to illustrate the agreement of accelerometer and GPAQ data regarding moderate and vigorous physical activity. In a Bland-Altman plot, the difference of two measures (y-axis) is plotted against the data of the accelerometer (x-axis) for each participant [33, 34]. The limits of agreement were set at mean difference ± 1.96 standard deviations. Plots were designed for moderate and vigorous physical activity of both GPAQ versions individually.

The significance level for all analyses was set at *p* < 0.05.

## Results

### Sample description

**Fig. 2:**
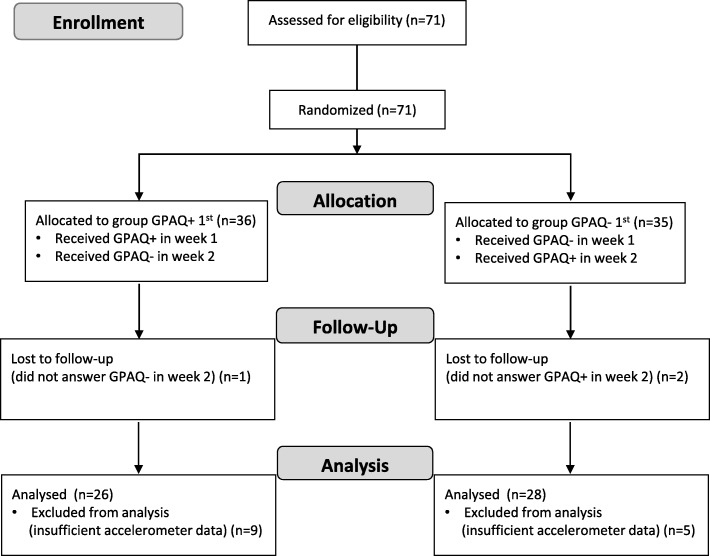
Flow chart of participation progress

All of the 71 recruited participants were eligible and provided written informed consent. A total of 17 participants were later excluded due to missing data. As a result, data of 54 participants (57.4% female, mean age: 28.3 ± 12.2 years; mean BMI: 23.2 ± 3.1 kg/m^2^; see Table 1) were analysed. Figure 2 shows the CONSORT flow chart illustrating the progress through the phases of the present study.

**Table 1:** Sample characteristics

	Total sample	Group GPAQ+ 1st	Group GPAQ- 1st	*p*
Sex [female] n (%)	31 (57.4)	17 (65.4)	14 (50.0)	0.25^1^
Age [years] mean (SD)	28.3 (12.2)	26.1 (8.9)	30.3 (14.6)	0.34^2^
BMI [kg/m^2^) mean (SD)	23.2 (3.1)	23.0 (3.3)	23.3 (3.0)	0.51^2^

^**1**^ Chi-squared test; ^2^Mann-Whitney U test

No statistically significant differences were found between group GPAQ+ 1st and GPAQ- 1st at baseline regarding sex (*p* = 0.25), age (*p* = 0.34) or BMI (*p* = 0.51). People excluded from the study due to insufficient activity data did not show statistically significant differences to the analysed sample regarding age (*p* = 0.29) or BMI (*p* = 0.06), but were more likely to be men (*p* = 0.04).

Table 2 shows the daily averages of both GPAQ versions as well as data from the accelerometer measurement for the respective time frames. Regardless of operationalization, the sample shows high amounts of moderate to vigorous physical activity of more than 2 hours per day on average.

**Table 2:** Data on physical activity measured by GPAQ+, GPAQ-, and accelerometer

***n*** **=** **54**	**GPAQ +**	**Accelerometer**	**p**^**1**^
Moderate intensity [min/d] mean (SD)	86.8 (90.5)	109.9 (29.7)	0.01*
Vigorous intensity [min/d] mean (SD)	48.7 (47.9)	13.4 (11.3)	< 0.01*
Sedentary behaviour [min/d] mean (SD)	420.8 (154.2)	439.2 (78.5)	0.61
***n*** **=** **54**	**GPAQ -**	**Accelerometer**	**p**^**1**^
Moderate intensity [min/d] mean (SD)	88.6 (121.0)	109.9 (33.1)	< 0.01*
Vigorous intensity [min/d] mean (SD)	42.4 (36.1)	12.1 (9.4)	< 0.01*
Sedentary behaviour [min/d] mean (SD)	426.9 (163.3)	436.2 (81.6)	0.83

^**1**^ Wilcoxon signed-rank tests; *statistically significant (*p* < 0.05)

While no statistically significant differences were found for data on sedentary behaviour, moderate and vigorous intensity data measured by both GPAQ versions differ statistically significant from accelerometer data (all *p* < 0.05; see Table 2).

### Comparison and association of questionnaire and accelerometer data

In line with the comparable mean values shown in Table 2, no statistically significant differences could be found between GPAQ+ and GPAQ- for any intensity of physical activity regarding the differences to the accelerometer data (see Table 3; all *p* > 0.05).

**Table 3:** Mean differences of both GPAQ versions to accelerometer data

*n* = 54	Difference: GPAQ+ to accelerometer	Difference: GPAQ- to accelerometer	p^1^
Moderate intensity [min/d] mean (SD)	−23.1 (89.3)	−21.3 (117.1)	0.33
Vigorous intensity [min/d] mean (SD)	35.2 (45.5)	30.3 (35.4)	0.40
Sedentary behaviour [min/d] mean (SD)	−18.3 (150.7)	−9.3 (159.6)	0.32

^**1**^ Wilcoxon signed-rank tests

Statistically significant fair correlations were present between GPAQ+ and accelerometer data regarding sedentary behaviour (rho = 0.32; *p* = 0.02) and vigorous physical activity (rho = 0.42; *p* < 0.01). For GPAQ- and accelerometer data, results were similar regarding sedentary behaviour (rho = 0.29; *p* = 0.03) and vigorous physical activity (rho = 0.31; *p* = 0.02). In both questionnaire versions, no statistically significant correlations with accelerometer data were found for physical activity of moderate intensity (GPAQ+: rho = 0.19; *p* = 0.17; GPAQ-: rho = 0.18; *p* = 0.20).

**Fig. 3:**
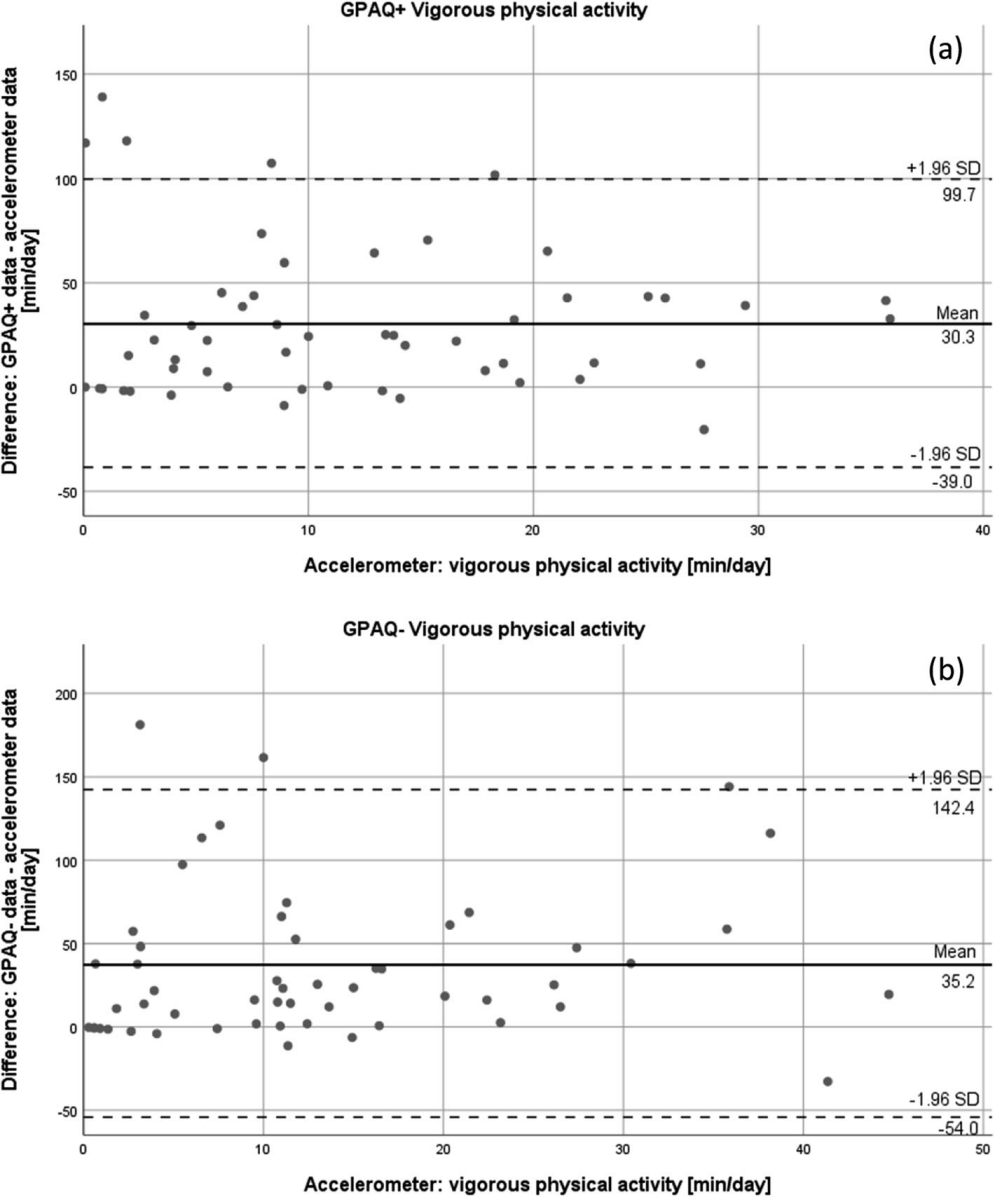
Bland-Altman plots for vigorous physical activity for (**a**) GPAQ+ and (**b**) GPAQ-

**Fig. 4:**
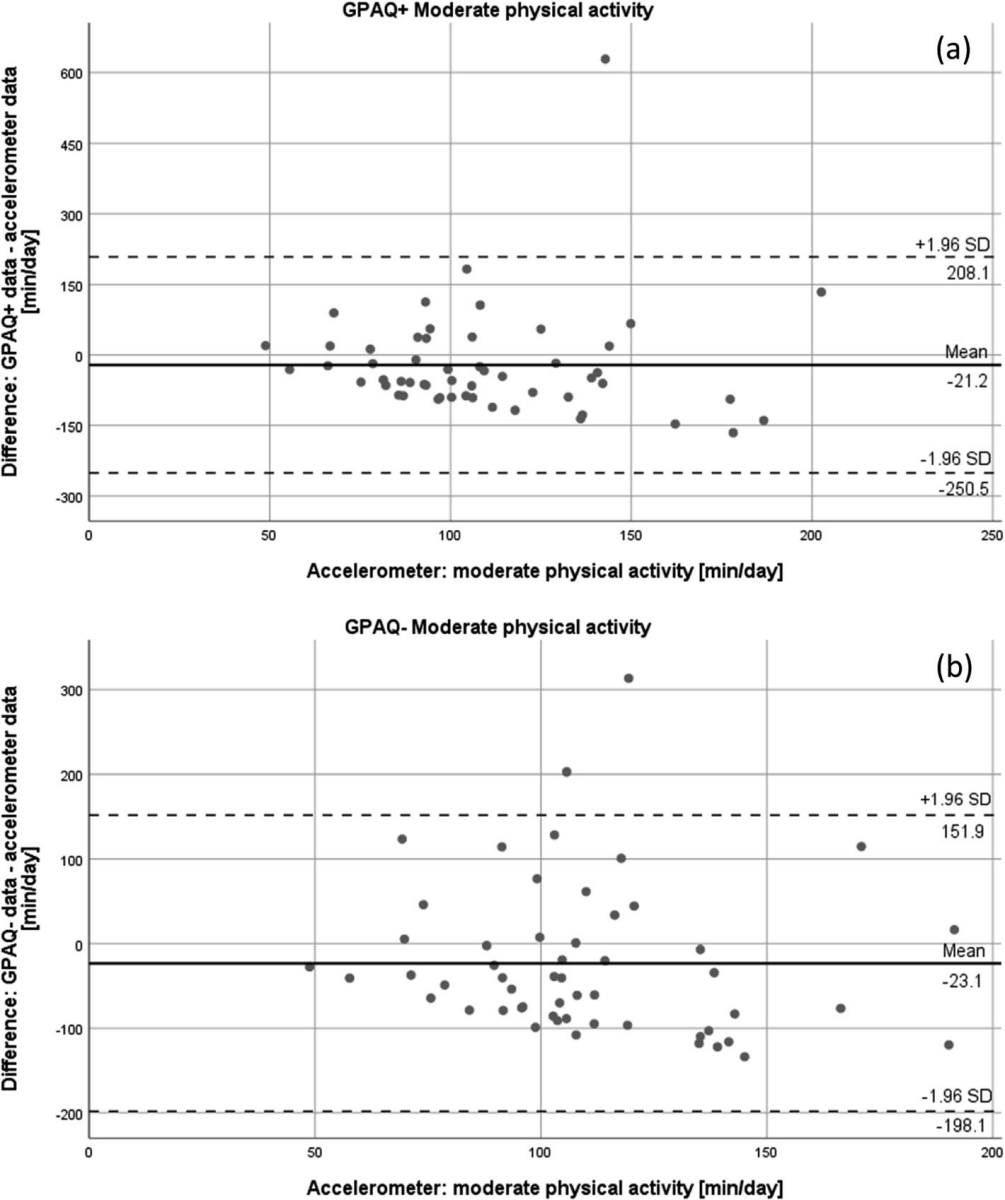
Bland Altman plots for moderate physical activity for (**a**) GPAQ+ and (**b**) GPAQ-

**Fig. 5:**
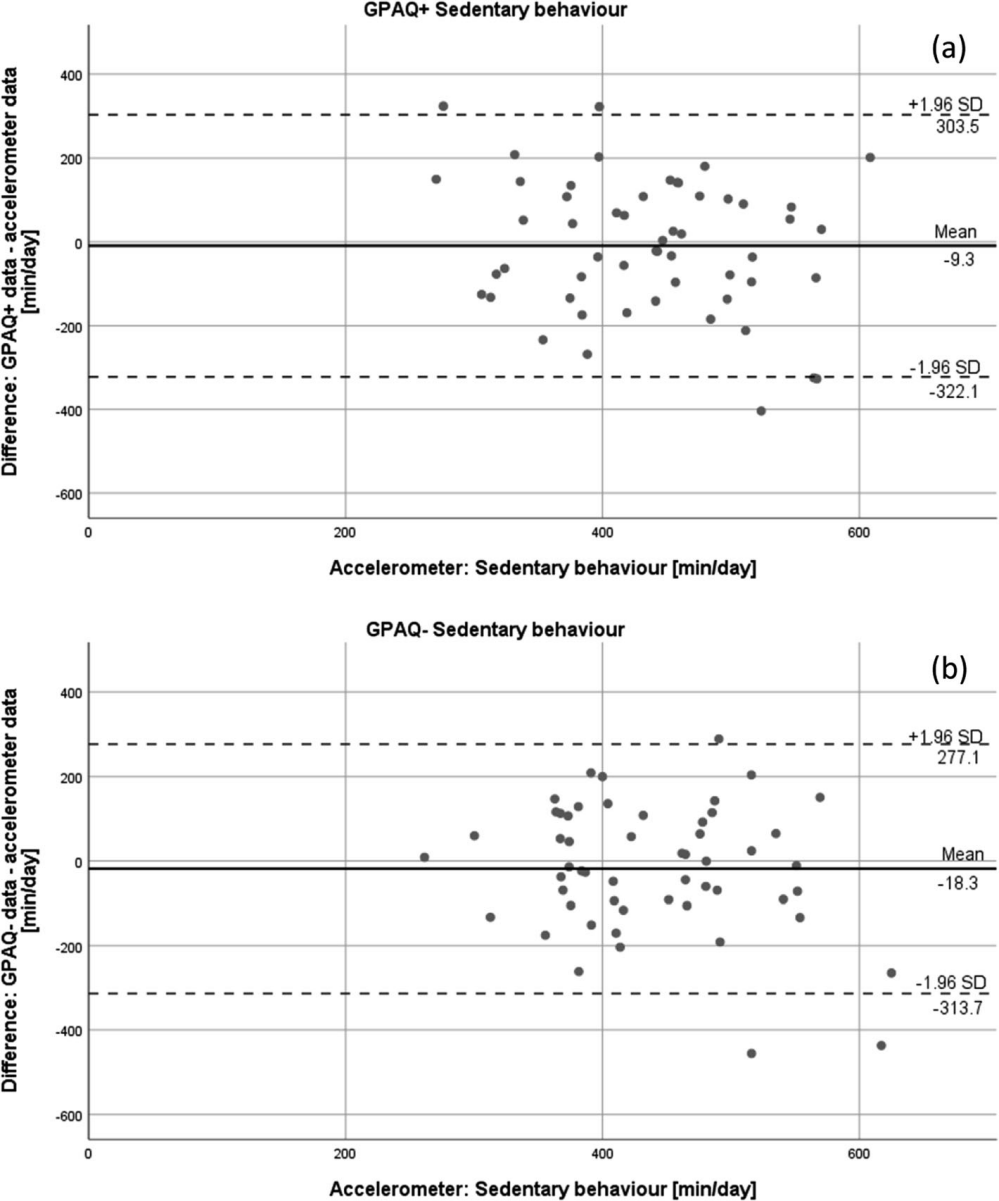
Bland-Altman plots for sedentary behaviour for (**a**) GPAQ+ and (**b**) GPAQ-

Figures 3, 4, and 5 show the Bland-Altman plots for the agreement of both GPAQ versions and accelerometer data for vigorous (Fig. 3) and moderate (Fig. 4) physical activity and sedentary behaviour (Fig. 5). The plots illustrate an average over-reporting of vigorous physical activity of 30.3 (±35.4) minutes per day in the GPAQ+ version and 35.2 (±45.5) minutes per day in the GPAQ- version in comparison to the accelerometer data. Moderate physical activity, on the other hand, was mostly under-reported with a mean of 21.2 (±117.1) and 23.1 (±89.3) minutes per day for GPAQ+ and GPAQ-, respectively. Although the mean differences are small, the wide limits of agreement indicate bigger discrepancies between GPAQ and accelerometer data on an individual level. The same applies for the data on sedentary behaviour. While the mean differences between GPAQ and accelerometer data for sedentary time are quite small (GPAQ-: − 18.3 ± 150.7 min/day; GPAQ+: − 9.3 ± 159.6 min/day), the limits of agreement are wide as well.

## Discussion

### Interpretation

To our knowledge, the present study is the first examining possible differences in validity of the GPAQ depending on the application of show cards. Comparing the questionnaire data to simultaneously recorded accelerometer data, similar values of validity could be found. While the data show similar fair correlations of both GPAQ versions to the respective data from the accelerometry, no statistically significant associations are present for physical activity of moderate intensity. In addition, Bland-Altman plots and Wilcoxon signed-rank tests show similar patterns of over- and under-reporting of physical activity in both GPAQ versions.

Overall, the results of the present study show that the application of the GPAQ without show cards does not inflict validity in the current sample. Whether or not show cards were used, the correlation of the GPAQ and accelerometer data was fair. This result goes in line with previous validation studies of the GPAQ that show poor to fair correlation to accelerometry, as well [18, 19, 35]. Hence, the use of the GPAQ without show cards deems equally valid as the application with show cards. This finding improves the feasibility of the GPAQ since the preparation and cultural adaption of show cards seems not to be necessary anymore. Moreover, it indicates that the results of previously conducted studies which did not use or report the use of show cards can be compared with studies which used the show cards.

Although the mean differences to the accelerometer data were relatively small, the differences on an individual level were very high for some participants who over- or underestimated their moderate to vigorous physical activity by more than 2 hours a day. This goes in line with previous studies which showed that overestimation of physical activity [36–38] and underestimation of sedentary behaviour [39, 40] are a big challenge in subjective physical activity measurements. In our study, a statistically significant under-reporting of daily moderate physical activity of about 21–23 min was present, as well as a statistically significant over-reporting of daily vigorous physical activity of about 30–35 min. Extrapolated to a week, the higher estimates of vigorous physical activity are more crucial than the under-reporting of moderate physical activity since already 75 min of vigorous physical activity a week are enough to achieve the recommended amount of physical activity [41]. Although the majority of the present sample was achieving the recommendations regardless of this discrepancy, these results hint at potential challenges within other, more inactive samples. The additional show cards are intended to show examples of physical activity and in this way help people to identify relevant behaviour and accurately estimate the intensity of physical activity. Since no difference for the application of show cards was found, one might argue that the problem does not lay within the physical activity intensities but in the estimation of the respective time frames [42]. Although the questionnaires ask for uninterrupted activities that lasts for at least 10 minutes, it might be hard for respondents to estimate the duration of activities that have breaks in it. As an example, one might say, that a person who engages in soccer for 90 min is physically active for 90 min as well. However, due to breaks within the game (fouls, throw-ins, corners etc.), the duration of physical activity normally does not exceed 60 min [43]. An accelerometer on the other hand, would provide a more accurate estimation since it relies on proper acceleration signals and, hence, only tracks real movement. To improve accuracy of self-reports, further research should focus on the implication of phrases which inform participants about these problems. Additionally, for the field of sedentary behaviour research, phrases suggesting to calculate sedentary time by counting the waking hours and subtracting hours in which people do not engage in physical activity of at least light intensity, might be a possible way to go for.

### Strengths and limitations

A limitation of the present study is the composition of the convenience sample. Since recruitment was conducted mainly via bulletins in the German Sports University and several sports clubs, the resulting sample had a high level of physical activity. This could have an impact on the results since the sample might be more aware of the intensity of physical activity due to their sports background and, hence, does not need additional examples in form of show cards to assess intensity correctly. The question arises whether a less active sample would produce similar results or benefit more from the examples provided by the show cards.

Another limitation is the selection of activities depicted in the show cards. Although we chose activities by using the” Compendium of Physical Activities “[25], work situations and leisure time activities are manifold and obviously not all of them could be depicted due to limited space. The formal classification may not reflect the individual performance and the resulting physical strain while performing the activity [42]. A soccer match can be of vigorous or moderate intensity depending on the individual’s engagement and position within the game (e.g., goal keeper vs. midfielder). Hence, participants still had to compare their activities with those depicted in the show cards, possibly resulting in misclassification intensity-wise.

A strength of the current study is the crossover design. Due to the two groups answering the GPAQ+ and GPAQ- in different weeks, effects of test repetition and sequence-effects were ruled out. Moreover, the simultaneous application of the accelerometer can be seen as an additional strength of the study. By comparing the questionnaires in terms of validity against accelerometry, we collected more information on the similarities between the GPAQ versions than by just filling out each questionnaire. In this way, the current study provides comprehensive validity data for the application of the GPAQ with and without show cards. However, the processing of the Actigraph GT3X+ data inherits possible limitations, as well. Since the conversion of the CPM into activity data relies on special cut-points, it cannot be ruled out that the application of other cut-points might result in different amounts of moderate and vigorous physical activity [44]. Cut-points, e.g., with lower CPM-thresholds for vigorous physical activity would possibly result in less under-reporting of moderate physical activity and less over-reporting of vigorous physical activity. However, we used the cut-points of Freedson et al. [30] since they are widely used in the existing literature and most of the other frequently used cut-points for adults inherit very similar thresholds for vigorous physical activity [45].

## Conclusion

The measurement of physical activity for scientific purposes is a challenge that moves between two poles, validity and feasibility. The current study shows that the application of the GPAQ without show cards is more practicable and has no negative effect on the questionnaire’s validity in the target sample. Hence, the GPAQ can be used without show cards in practice-oriented research in young, healthy and physically active samples. Further research with different and less active target groups is needed.

## Supplementary information

**Additional file 1.** Illustration of the GPAQ show cards.

**Additional file 2.** Illustration of the application of the accelerometer.

## Data Availability

The datasets used and analyzed during the current study are available from the corresponding author on reasonable request.

## Abbreviations

BMI: Body mass index
CPM: Counts per minute
DLW: Doubly-labeled water method
GPAQ: Global Physical Activity Questionnaire
GPAQ: Global Physical Activity Questionnaire without show cards
GPAQ+: Global Physical Activity Questionnaire with show cards
MET: Metabolic equivalent of task
STEPS: STEPwise approach for surveillance of risk factors for chronic disease
WHO: World Health Organization
