# Supplementary material for: Show cards of the Global Physical Activity Questionnaire (GPAQ) – do they impact validity? A crossover study
Source: BMC Public Health. 2020 Feb 12;20:223. doi: 10.1186/s12889-020-8312-x (PMC7017628; doi:10.1186/s12889-020-8312-x)
Supplement: Supplementary file 1 — Additional file 1. Illustration of the GPAQ show cards. [file 12889_2020_8312_MOESM1_ESM.pdf]

Vigorous Physical Activity at Work

Examples for vigorous activities at WORK

VIGOROUS Intensity Activities

Make you breathe <u>much</u> harder than normal

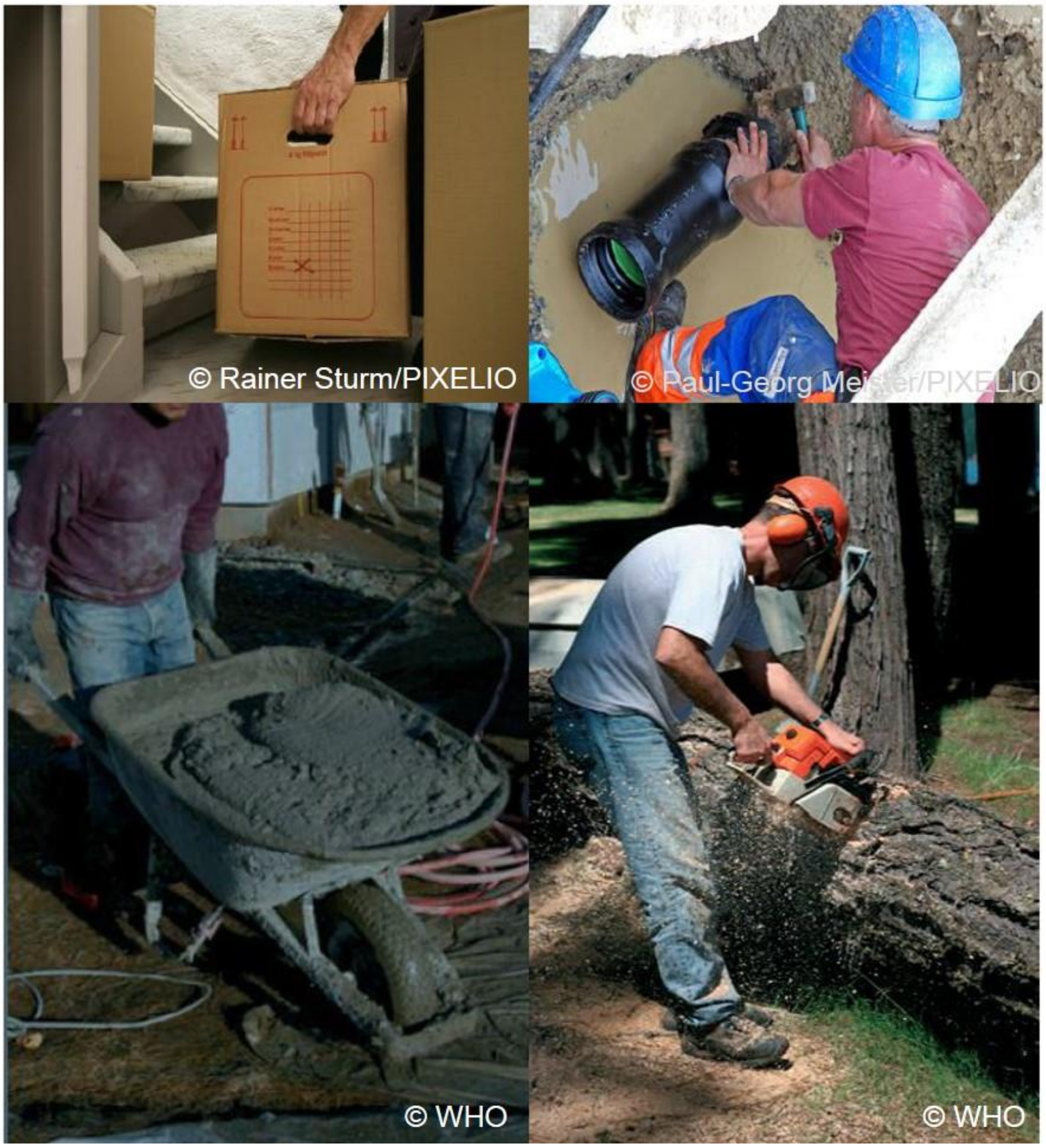

Picture credit: original version of the GPAQ (WHO) and www.pixelio.de.

Other examples for VIGOROUS activities at WORK

- Gardening (digging)
- Steel worker (molten steel)
- Fitness instructor (spinning, aerobics)
- Forestry (cutting, chopping, carrying wood)
- Firefighter (in action)

Moderate Physical Activity at Work

Examples for MODERATE activities at work

MODERATE Intensity Activities
Make you breathe <u>somewhat</u> harder than normal

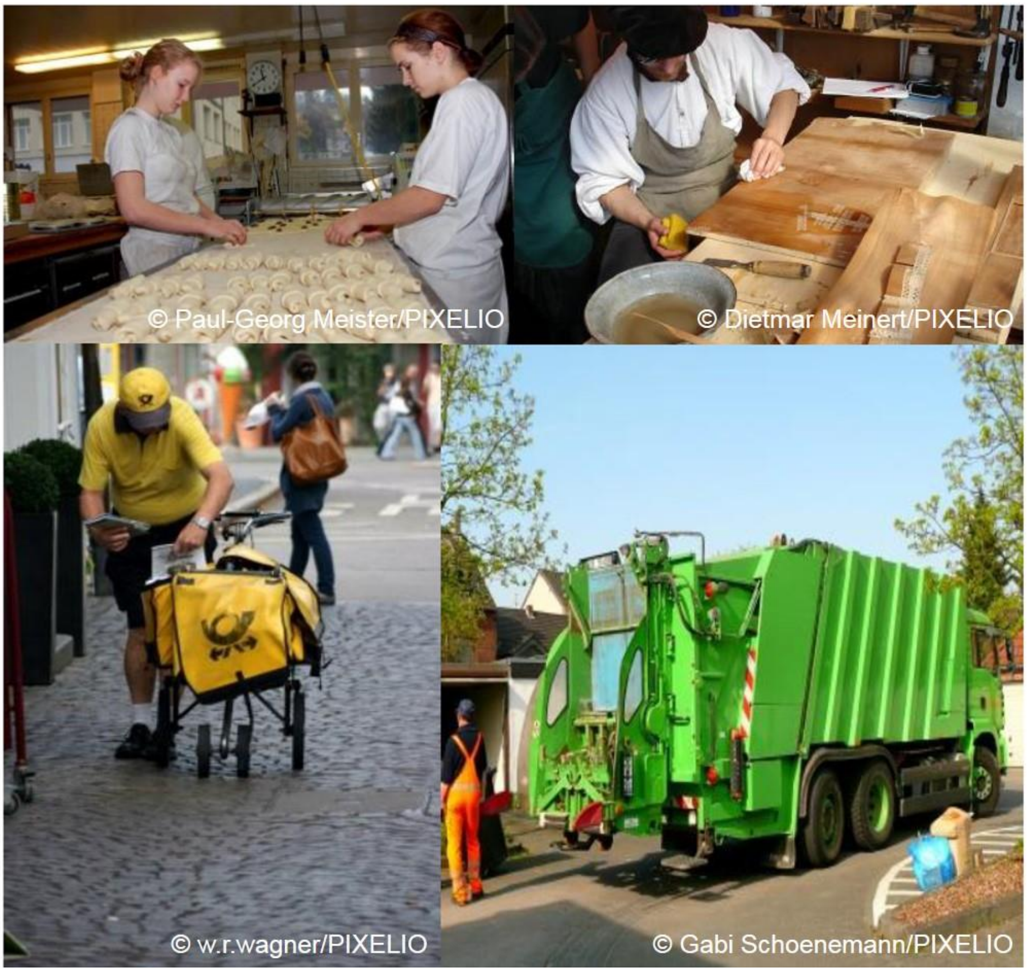

Picture credit: original version of the GPAQ (WHO) and www.pixelio.de.

Other examples for MODERATE activities at WORK

- Working on the assembly line (industry)
- Mining (coal minining)
- Farming (tending animals, planting and harvesting crops)
- Mixing cement (with shovel)
- Stewardess (pushing carts)
- Labouring (pushing loaded wheelbarrow, operating jackhammer)

Vigorous Physical Activity during Leisure Time

Examples for VIGOROUS activities during LEISURE TIME

VIGOROUS Intensity Activities
Make you breathe much harder than normal

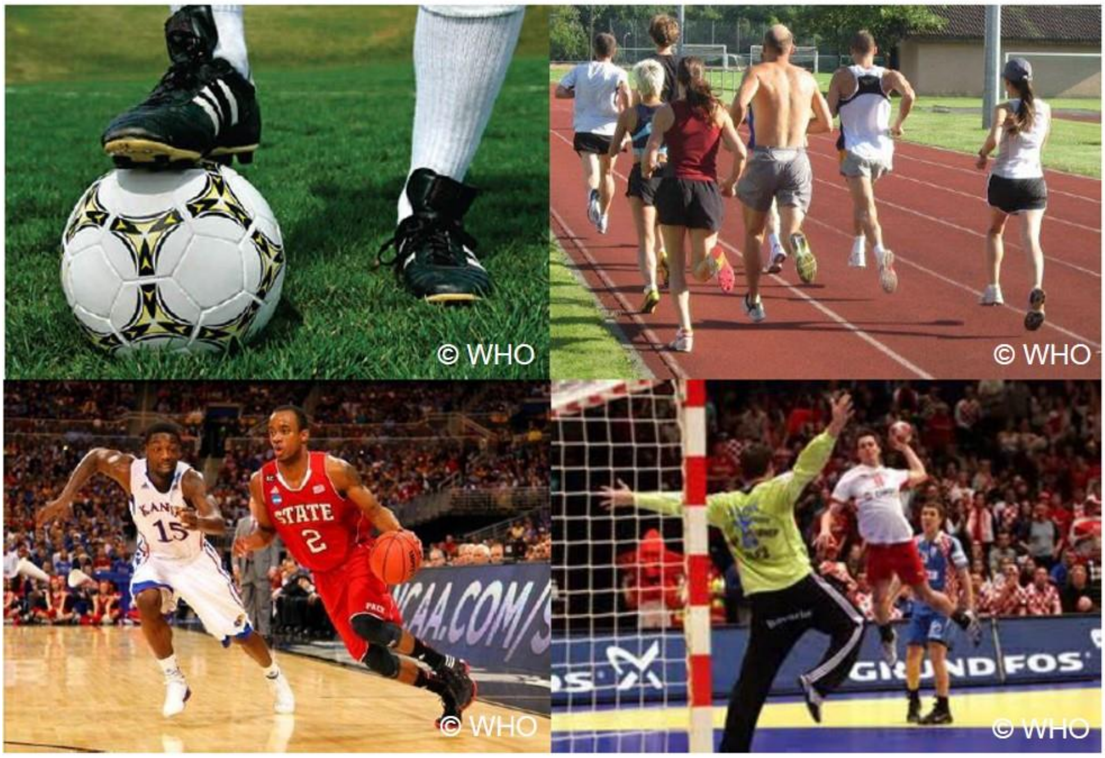

Picture credit: original version of the GPAQ (WHO).

Other examples for VIGOROUS activities during LEISURE TIME

- Tennis
- Aqua aerobics
- Ballet dancing
- Ice hockey
- Show jumping
- Football
- Boxing
- Judo/ Karate
- Squash
- Rock climbing
- Rugby

Moderate Physical Activity during Leisure Time

Examples for MODERATE activities during LEISURE TIME

MODERATE Intensity Activities

Make you breathe somewhat harder than normal

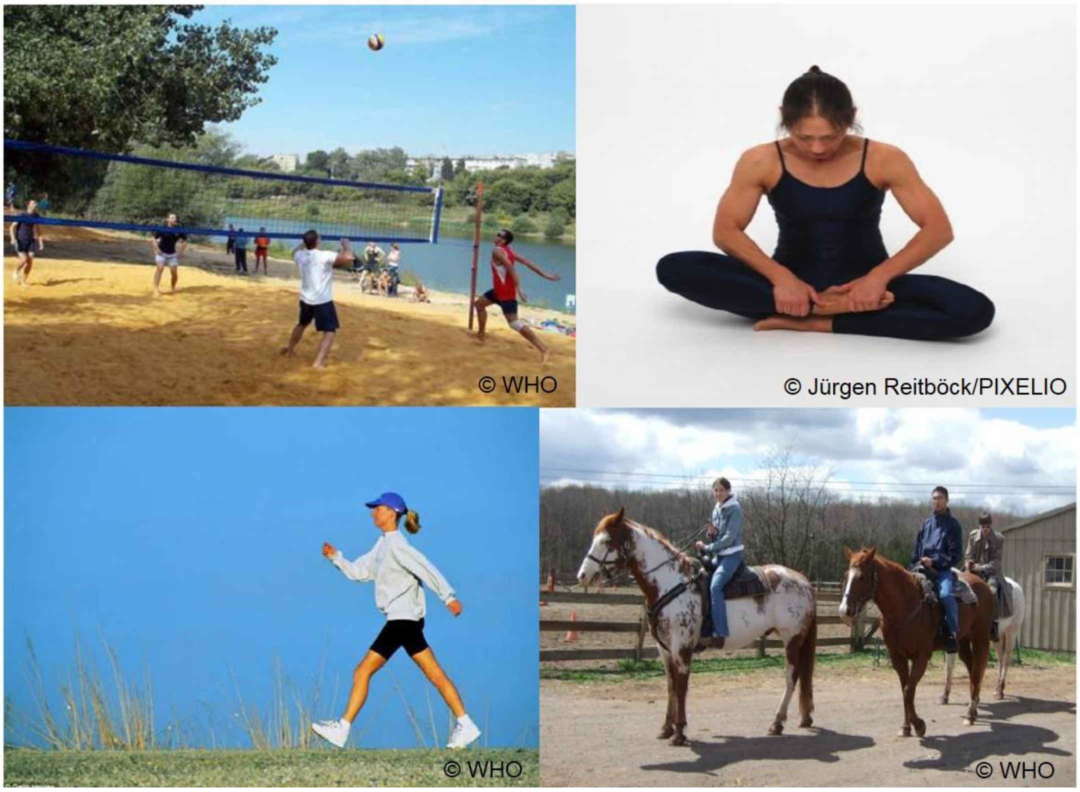

Picture credit: original version of the GPAQ (WHO) and www.pixelio.de.

Other examples for MODERATE activities at WORK

- Jogging
- Cycling
- Dancing
- Table tennis
- Golf
- Bowling
- Pilates
- Tai chi
