# Supplementary figures and images for: Show cards of the Global Physical Activity Questionnaire (GPAQ) – do they impact validity? A crossover study
Source: BMC Public Health. 2020 Feb 12;20:223. doi: 10.1186/s12889-020-8312-x (PMC7017628; doi:10.1186/s12889-020-8312-x)

**Picture of the Actigraph GT3X+**

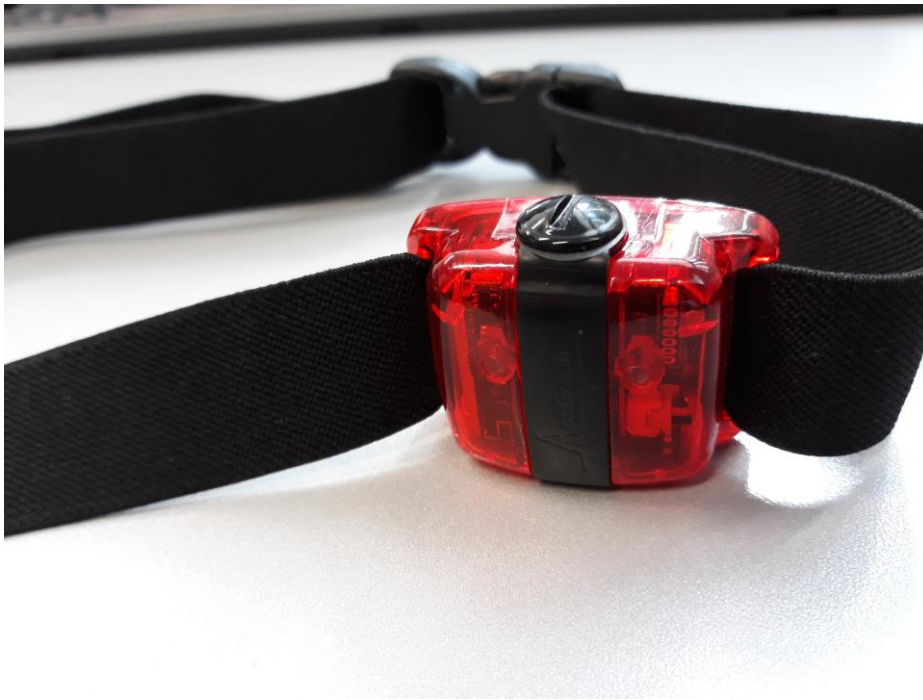

**Picture of the application of the Actigraph GT3X+**

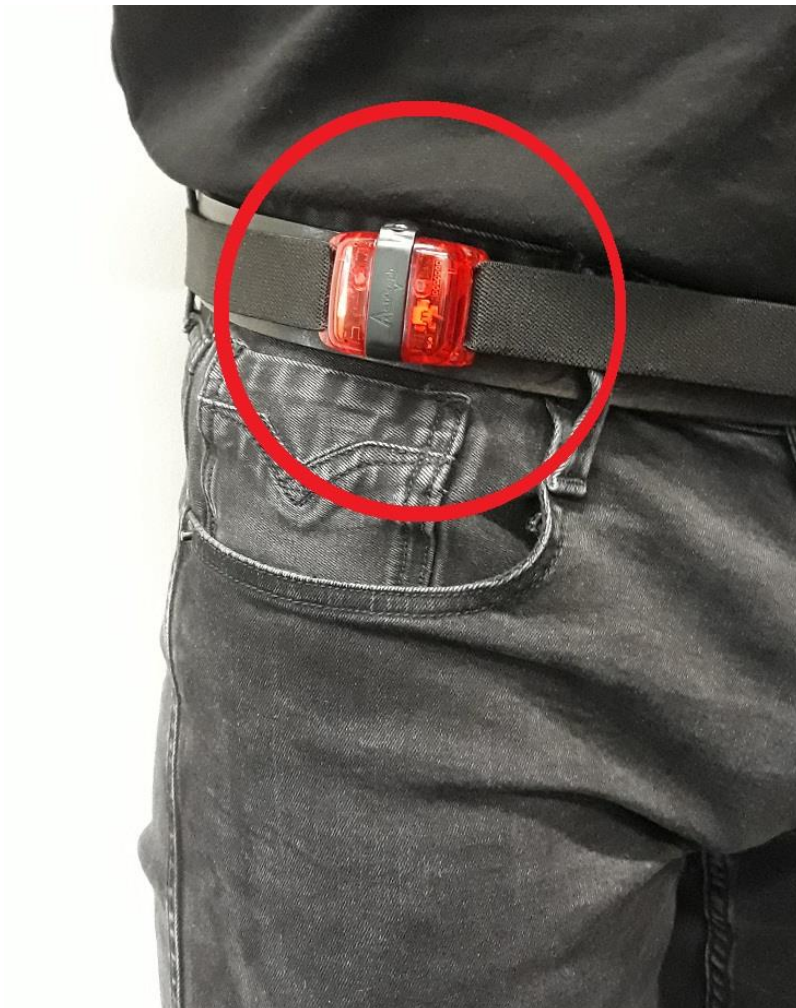

Supplement: Supplementary file 2 — Additional file 2. Illustration of the application of the accelerometer. [file 12889_2020_8312_MOESM2_ESM.pdf]
